# Shelby Medical College, Nashville, Tenn.

**Published:** 1861-08

**Authors:** 


### Shelby Medical College, Nashville, Tennessee.

We are requested to publish the following announcement of the above Institution. Shelby Medical College, though of recent organization, has made a highly favorable impression upon the profession at large, and enjoys a deservedly high reputation with the Colleges of our country. We are personally acquainted with some of its Faculty, and know them to be high-toned gentlemen—men of talents and learning—and hope ere long to hear that that they have resumed their Collegiate labors, with a large class of pupils:

"*Announcement.*—In consequence of the existing war between the governments of the Confederate States of America and the United States of America—the proximity of this point to the territorial line dividing the combatants—the possibility that this vicinity may, in the course of the ensuing six or twelve months, become the theatre of hostile operations—the excited condition of the public mind generally, and especially among that class of the community yielding pupils to institutions of medical learning—the unfitness for receiving instruction, either satisfactory or profitable, which necessarily results from this state of mind—the large number of the youth of the country who are already in the military service—the stringency everywhere prevailing in regard to pecuniary matters caused by the posture of affairs above described—and the fact that *four* of our corps of teachers are employed in professional positions connected with the army, the Faculty of Shelby Medical College have deemed it proper to announce that the Winter course of Lectures for 1861–62 will not be opened, and that this suspension of exercises will be continued until further notice.

"JOHN H. CALLENDER, M.D., Prof. Therapeutics, etc.
"THOMAS L. MADDIN, M.D., Prof. Surgery.
"DANIEL B. CLIFFE, M.D., Prof. Anatomy.
"J. J. ABERNATHY, M.D., Prof. Theory and Practice.
"DANIEL F. WRIGHT, M. D., Prof. Physiology and Pathology.
"HENRI ERNI, M.D., Prof. Chemistry.
"JOHN P. FORD, M.D., Prof. Obstetrics, etc., and Dean of the Faculty.
"H. M. COMPTON, M.D., Demonstrator of Anatomy."

*Nashville*, Aug. 22, 1861.

